# Enhancing the sample diversity of snowball samples: Recommendations from a research project on anti-dam movements in Southeast Asia

**DOI:** 10.1371/journal.pone.0201710

**Published:** 2018-08-22

**Authors:** Julian Kirchherr, Katrina Charles

**Affiliations:** 1 Faculty of Geosciences, Utrecht University, Utrecht, the Netherlands; 2 School of Geography and the Environment, University of Oxford, Oxford, United Kingdom; University of Michigan, UNITED STATES

## Abstract

Snowball sampling is a commonly employed sampling method in qualitative research; however, the diversity of samples generated via this method has repeatedly been questioned. Scholars have posited several anecdotally based recommendations for enhancing the diversity of snowball samples. In this study, we performed the first quantitative, medium-*N* analysis of snowball sampling to identify pathways to sample diversity, analysing 211 reach-outs conducted via snowball sampling, resulting in 81 interviews; these interviews were administered between April and August 2015 for a research project on anti-dam movements in Southeast Asia. Based upon this analysis, we were able to refine and enhance the previous recommendations (e.g., showcasing novel evidence on the value of multiple seeds or face-to-face interviews). This paper may thus be of particular interest to scholars employing or intending to employ snowball sampling.

## Introduction

Snowball sampling is a commonly employed sampling method in qualitative research, used in medical science and in various social sciences, including sociology, political science, anthropology and human geography [1–3]. As is typical of terms adopted by a variety of fields, however, the phrase ‘snowball sampling’ is used inconsistently across disciplines [4]. The most frequently employed definition, suggested by Patton [5], Atkinson and Flint [6], Cohen and Arieli [7] and Bhattacherjee [8], is as a sampling method in which one interviewee gives the researcher the name of at least one more potential interviewee. That interviewee, in turn, provides the name of at least one more potential interviewee, and so on, with the sample growing like a rolling snowball if more than one referral per interviewee is provided.

This definition can initially seem self-explanatory, which may explain why snowball sampling is rarely discussed in most peer-reviewed papers that employ it. Various scholars use snowball sampling in their empirical work, but most provide only limited information on the method (see, e.g., [9–13]). Similarly, qualitative research textbooks often lack substantive discussion of snowball sampling (e.g., [8, 14–19]). Bailey [14], for instance, devotes only a half-page of his 595-page book on social research methods to snowball sampling, acknowledging that ‘snowball sampling procedures have been rather loosely codified’ ([14], p. 96), an observation echoed by Penrod et al. [3].

This paper focuses on snowball sampling procedures, which we define as those actions undertaken to initiate, progress and terminate the snowball sample [1, 20]. Despite the lack of substantive writing on snowball sampling as a method, several authors [2, 3, 21] have provided recommendations for enhancing a sample’s diversity in snowball sampling procedures (we discuss this further in Section 4). However, as this advice is not based on a quantitative analysis of evidence, but only on anecdotal evidence, there is a risk that these recommendations are based on coincidence. The aim of this paper is to provide advice on enhancing the sample diversity of a snowball sample. This advice is grounded in a medium-*N* analysis of relevant evidence, thus reducing the probability of positing advice that is based on coincidence [22]. A medium-*N* analysis is generally based on 10–100 cases, whereas anecdotal evidence is usually based only on a handful of cases [23, 24]. At the core of our work, we provide descriptive analyses of various commonly prescribed strategies for enhancing the sample diversity of a snowball sample. These analyses are based on reach-outs to 211 individuals via snowball sampling for a research project on anti-dam movements in Southeast Asia, resulting in 81 interviews conducted between April and August 2015. As far as we are aware, ours is the first medium-*N* analysis to focus on enhancing the sample diversity of a snowball sample.

The remainder of this paper is organised as follows: in Section 2, we discuss snowball sampling as a method; in Section 3, we present the research project on anti-dam movements in Southeast Asia that served as the basis for our medium-*N* analysis on snowball sampling procedures; in Section 4, we present and discuss insights on snowball sampling procedures based upon this analysis as well as our resulting recommendations; finally, in Section 5, we summarise our argument.

Throughout this paper, we employ social science methodology terminology. We define key terms for this paper such as ‘snowball sampling’ or ‘sampling’, since these terms are not consistently codified in the scholarly literature. Due to limited space, however, we refrain from defining terms we have deemed common in this field of study, referring only to the relevant literature.

## On snowball sampling

Traditional sampling methods are comprised of two elements [25, 26]. First, a full set of data sources is defined, creating a list of the members of the population to be studied, known as a sampling frame. Second, a specific sample of data is collected from this sampling frame. Snowball sampling defies both elements, since it does not rely upon a sampling frame [27] (which may indicate that a different term for snowball sampling would be more accurate). Snowball sampling is often employed when no sampling frame can be constructed.

Researchers frequently cannot construct a sampling frame if a difficult-to-reach population is to be studied. Difficult-to-reach-populations are also referred to as ‘hard-to-reach-populations’ [28], ‘hidden populations’ [29] or ‘concealed populations’ [21] in the scholarly literature. Although not all scholars may agree that these terms are interchangeable, we deem them interchangeable for the purposes of this paper. For further discussion of this terminology, see [30, 31].

A difficult-to-reach population does not wish to be found or contacted (e.g., illegal drug users, illegal migrants, prostitutes or homeless people [6, 31]). Snowball sampling was originally used by researchers to study the structure of social networks [32]. The earliest empirical account of snowball sampling is from 1955 [33], with snowball sampling first described as a method in 1958 [34]. While it is still used to study the structure of social networks [35], over the last few decades, the method’s key purpose has largely transformed ‘into […] an expedient for locating members of a [difficult-to-reach] population’ ([36], p. 141).

Researchers grounded in quantitative thinking, such as Lijphart [37] and King et al. [38], tend to view the drawing of a random sample from a sampling frame as the gold standard of data collection. Even these researchers may nevertheless consider non-probability sampling methods, such as snowball sampling, a ‘necessary and irreplaceable sampling [method]’ ([39], p. 367) when confronted with difficult-to-reach populations, particularly if the dismissal of snowball sampling would mean that no research could be conducted at all. Ultimately, ‘an important topic is worth studying even if very little [access to] information is available’ ([38], p. 6). Still, some of those grounded in quantitative thinking call snowball sampling a method ‘at the margin of research practice’ ([6], p. 1), since the lack of a sampling frame means that, unlike individuals in a random sample, individuals in a population of interest do not have the same probability of being included in the final sample. Findings from a snowball sample would therefore not be generalisable [40] (on generalisability, see [41]).

Several qualitative scholars rebut such criticism. Creswell, for instance, notes that ‘the intent [of qualitative research] is not to generalise to a population, but to develop an in-depth [and contextualised] exploration of a central phenomenon’ ([42], p. 203). Others [1, 39] specifically oppose quantitative scholars’ negative framing of snowball sampling, arguing that this method would ‘generate a unique type of social knowledge’ ([1], p. 327). Due to the diversity of perspectives gathered, this knowledge would be particularly valuable for an in-depth and contextualised exploration of a central phenomenon. We therefore define the diversity of a sample as a measure of the range of viewpoints that have been gathered on a central phenomenon.

Researchers critical of snowball sampling respond to this defence by arguing that the method is unable to ensure sample diversity, which is a necessary condition for valid research findings. Indeed, some scholars have stated that snowball samples underrepresent and may even exclude those least keen to cooperate, since referrals may not materialise in an interview if a potential interviewee is only somewhat keen or not at all keen to be interviewed [3, 43]. Similarly, potential interviewees with smaller networks may be underrepresented, as they are less likely to be referred for an interview [31, 44]. Those with smaller networks may also be in a specific network whose different perspectives may be of interest but are excluded in the final sample. Meanwhile, snowball sampling is said to over represent those interviewees (and their respective networks) that the interviewer spoke with first; the relevant literature refers to this as ‘anchoring’ [20, 39].

We do not aim to argue the ‘validity’ of the method, but rather to inform snowball sampling methodologies in order to promote sample diversity. From a qualitative perspective, ‘validity’ can be defined as ‘the correctness or credibility of a description, conclusion, explanation, interpretation or other sort of account’ ([45], p. 87), while quantitative researchers frequently use the terms ‘generalisability’ and ‘(external) validity’ interchangeably [46, 47]. The term ‘validity’ is contested among qualitative researchers, and some qualitative researchers entirely reject the concept for qualitative work [48, 49]. We do not aim to resolve this debate via this paper; instead, we focus on the (seemingly less-contested) term ‘sample diversity’. While we acknowledge that this term is not codified in qualitative textbooks such as the *SAGE Encyclopedia of Qualitative Research Methods*, sample diversity is considered desirable by the various qualitative scholars we reviewed. Boulton and Fitzpatrick demand, for instance, that qualitative researchers ‘ensure that the full diversity of individuals […] is included [in their sample]’ ([50], p. 84), a mandate echoed by other scholars [16, 51–53].

In order to operationalise the concept of sample diversity, we used five key methodological recommendations to inform our research. In this paper, we use quantitative analyses from our experiences with snowball sampling to further reflect on these recommendations, which are briefly described below.

### Prior personal contacts of the researcher are required

Patton ([5], p. 176) notes that snowball sampling ‘begins by asking well-situated people: “Who knows a lot about ____? Who should I talk to?”‘. In the absence of a sampling frame for the population of interest, however, the researcher must retain at least some prior personal or professional contacts in the population of interest which can serve as the seeds of the snowball sample [2, 54]. Waters contends that building a diverse snowball sample ‘depend[s] almost exclusively on the researcher’s [prior personal or professional] contacts’ ([39], p. 372).

### Sample seed diversity is important

Morgan [21] has claimed that the ‘best defence’ against a lack of sample diversity is to begin the sample with seeds that are as diverse as possible. Others echo this advice [3, 39, 55], arguing that it is ‘compulsory for the researcher to ensure that the initial set of respondents is sufficiently varied’ ([55], p. 55). The term ‘chain referral sampling’ has been used for snowball samples that are strategically built via multiple varying seeds [3].

### Technology means face-to-face interviews are no longer required

Some researchers have argued that face-to-face interviews are obsolete. For instance, over 25 years ago, it was claimed there were ‘no remarkable differences’ ([56], p. 211) between information collected via telephone and information collected via face-to-face interviews. The increasing use of telecommunications in recent years is likely to have further reduced barriers to remote interviewing, and various scholars [57,58] continue to claim that ‘evidence is lacking that [telephone interviews] produce lower quality data’ ([59], p. 391). In particular, they have highlighted the benefits of using Skype for semi-structured interviews [57].

However, for snowball sampling, face-to-face interviews help to generate the trust that scholars claim is required in order to gain referrals [1, 31, 39, 60]. Noy argues that ‘the quality of the referring process is naturally related to the quality of the interaction: […] if the researcher did not win the informant’s trust […], the chances the latter will supply the former referrals decrease’ ([1], p. 334).

### Persistence is necessary to secure interviews

Although the value of persistence may be considered self-evident by some scholars, it is seen by multiple academics [61–63] as a central virtue of qualitative researchers. Many young career scholars who embrace snowball sampling are likely to hear such advice as, ‘If you cannot interview your envisaged interviewees initially, don’t give up!’. A ‘helpful hint’ for qualitative researchers seeking informants is, ‘Persevere–repeat contact’ [64].

### More waves of sampling are required to access more reluctant interviewees

As a remedy for snowball sampling’s previously discussed bias towards excluding those least keen to be interviewed, multiple scholars suggest pursuing a snowball sample for multiple waves (with a new sampling wave reached once an interviewee introduces the interviewer to one or more potential interviewees) [65–68]. Those suggesting this remedy assume that pursuing more waves increases the likelihood of being referred to an interviewee from a particularly difficult-to-reach population who is at least somewhat keen to be interviewed.

## Methods

Approval for this study was granted by the Central University Research Ethics Committee (CUREC) of the University of Oxford. Our population of interest for our research project were stakeholders in Southeast Asia’s dam industry. Since ‘the most dramatic conflicts over how to pursue sustainable development’ ([69], p. 83) have occurred over the construction of large dams, we see this industry as a conflict environment with widely varying viewpoints. A conflict environment is one in which people perceive their goals and interests to be contradicted by the goals or interests of the opposing side [70]. The major conflicting parties in the dam industry tend to be local and international non-governmental organisations (NGOs) and academics (usually keen not to construct a particular dam) versus international donors, the private sector and governments (usually keen to construct a particular dam) [71, 72]. Each sub-population operating in a conflict environment can be considered difficult to reach since fear and mistrust are often pervasive [7]. Snowball sampling is a suitable research method in conflict environments because the introductions through trusted social networks that are at the core of this method can help interviewees to overcome fear and mistrust, which, in turn, ensures access [7]. This access is needed to gather the widely varying viewpoints in the hydropower industry, in particular viewpoints with regards to what constitutes just resettlement [73, 74]. Based on this rationale, we chose snowball sampling as the main method for our research.

In order to ensure sample diversity for our research project on anti-dam movements in Southeast Asia, we aimed to gather perspectives mostly from six main sub-populations: (1) local NGOs, (2) international NGOs, (3) international donors, (4) academia, (5) the private sector and (6) the government. We hypothesized that ‘dam developers’, a main sub-category of the interviewee category ‘private sector’, would be the most significant challenge to ensuring the diversity of our sample. Early in our process, many of the scholars with whom we discussed our research project argued that it would be impossible to interview a dam developer from a Chinese institution; meanwhile, researchers from a comparable research project that ended approximately when our project started reported being unable to interview any dam developers from European institutions. We also initially failed to collect data from dam developers: for instance, a survey we initiated that was distributed by Aqua~Media (host of a major global dam developer conference) to more than 1,500 dam developers yielded just five responses, only one of which was complete. We considered this weak response rate to be due, at least in part, to the dam industry’s negative view of academicians since the publication of Ansar et al. [75], which Nombre ([76], p. 1), the president of the International Commission on Large Dams (ICOLD), called ‘[highly] misleading’.

None of our researchers had significant direct links to the dam industry upon the start of the project; however, we did retain a variety of indirect links. Our researchers had past links to a management consultancy that serves various dam industry players, (more limited) links to an international donor working in the hydropower sector and links to activists in Myanmar advocating against dam projects.

After a favourable ethics review of our study by the CUREC of the University of Oxford, we commenced semi-structured interviews in April 2015, mostly via cold calls (we include cold e-mails in the term ‘cold calls’ throughout this paper). Initially, we conducted research via telephone only. We then undertook field research in Singapore, Myanmar and Thailand from June to August 2015 and terminated our data collection in late August 2015.

**Fig 1:**
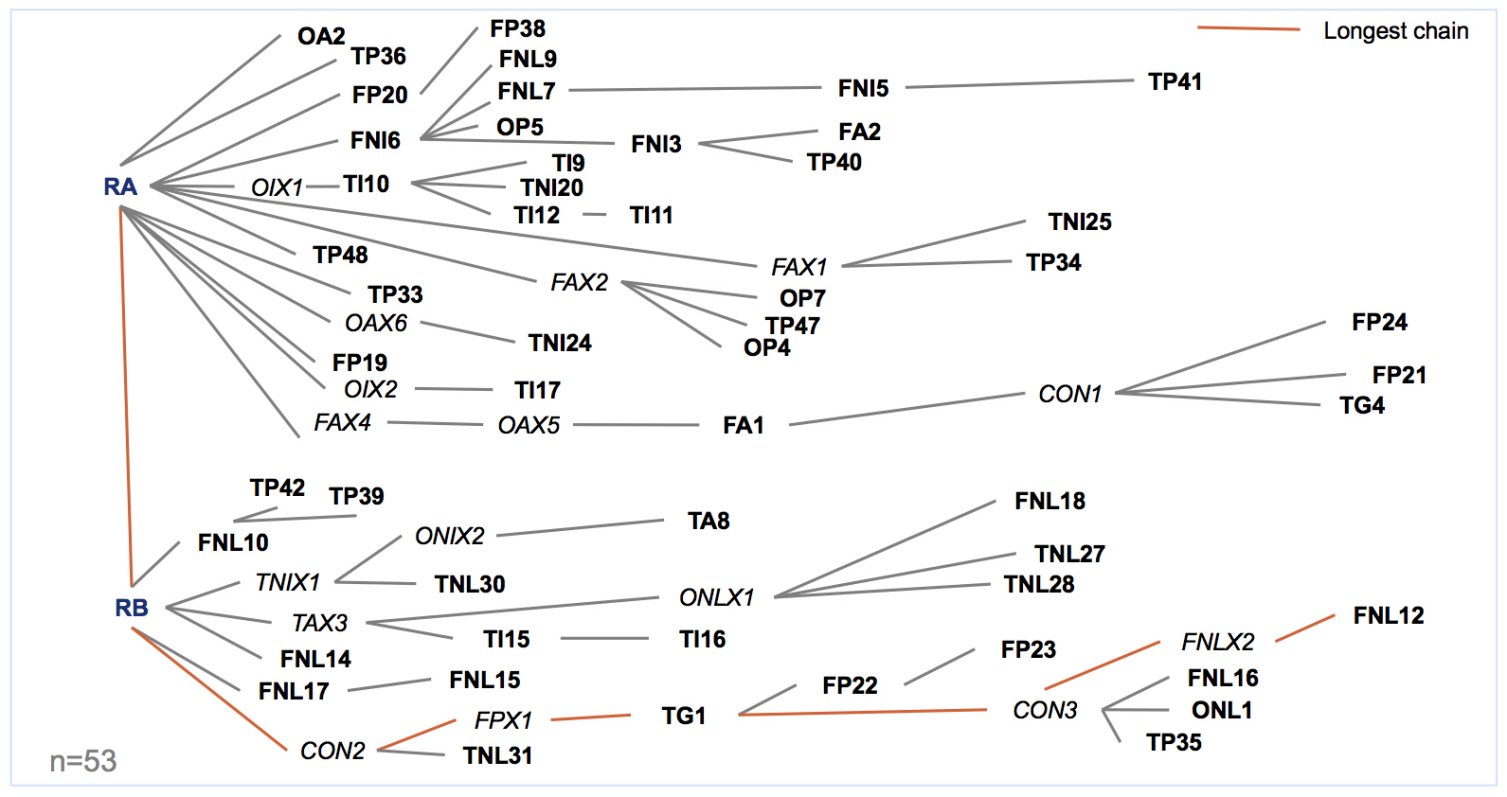
Recruitment network of snowball sample, starting with a single seed.

In total, 81 semi-structured interviews were carried out during this period. From a qualitative perspective, this is a relatively large sample size (for instance, the average qualitative PhD dissertation is based on 31 interviews [77]); from a quantitative perspective, however, the sample size is quite small [78]. Of our 81 interviews, 48 (59%) were conducted via telephone, 26 (32%) face-to-face and 7 (9%) online, either via e-mail or an online survey. Most of our interviews (57%) were carried out in July in Myanmar. Of our 81 interviewees, only 24 (30%) were women. Researchers who employ snowball sampling frequently employ personal/professional contact seeds and cold call seeds to build their sample (e.g., [2,79,80] with a seed defined as the starting point of a sample [65]). Of the 81 interviews analysed, 53 (65%) were rooted in a personal or professional contact (Fig 1) (i.e. the seed of the interview pathway was a contact we had already retained prior to the research project). The remaining 28 (35%) interviews were rooted in cold calls.

Given the sensitive nature of the interview topic, all interviewees were assured anonymity. Thus, all of the interviews are coded, with the first letter indicating the mode of interview (*T* for telephone, *F* for face-to-face, *O* for online survey or e-mail), the second letter indicating the category of interviewee (*A* for academia, *G* for government, *I* for international donor, *NI* for international NGO, *NL* for national NGO, *P* for private sector) and the sequence of numbers indicating the interview number within a particular mode. Researcher A is indicated by *RA*, Researcher B by *RB*; *CON* represents a conference event. Bold type indicates that an interview was completed, while *X* that an interview was not completed.

As outlined in the previous section, snowball sampling is sometimes criticised for producing samples that lack sample diversity. To address this criticism, we reviewed the (scarce) literature on enhancing sample diversity via snowball sampling procedures prior to commencing our study. Upon reflection during our research, we chose to pursue our analysis retrospectively in order to challenge some of the recommendations provided in literature. Our analysis is structured alongside the five core pieces of advice found in this literature (Table 1). Our results are based on a quantitative analysis of the 81 interviews we conducted. Although we endeavoured to include all interview attempts, some initial cold calls may have been overlooked in this retrospective approach. Therefore, some of our analysis, particularly in Section 4.4, may be too optimistic. Overall, we were able reconstruct 211 reach-out attempts.

**Table 1:** Summary of descriptive analyses. Sample diversity is measured by representation from five identified sub-groups.

Recommendation	Measure
Prior personal contacts of the researcher are required	Sample diversity within total interviews (and success of reach-outs) generated via cold calls compared with personal or professional contacts
Sample seed diversity is important	Sample diversity compared to initial seed
Technology means face-to-face interviews are no longer required	Comparison of referrals from telephone interviews with face-to-face overall, and by sample diversity
Persistence is necessary to secure interviews	Reach-outs to contacts per completed interview
More waves of sampling are required to access more reluctant interviewees	Sample diversity by wave

## Results and discussion

### On prior personal and professional contacts

**Fig 2:**
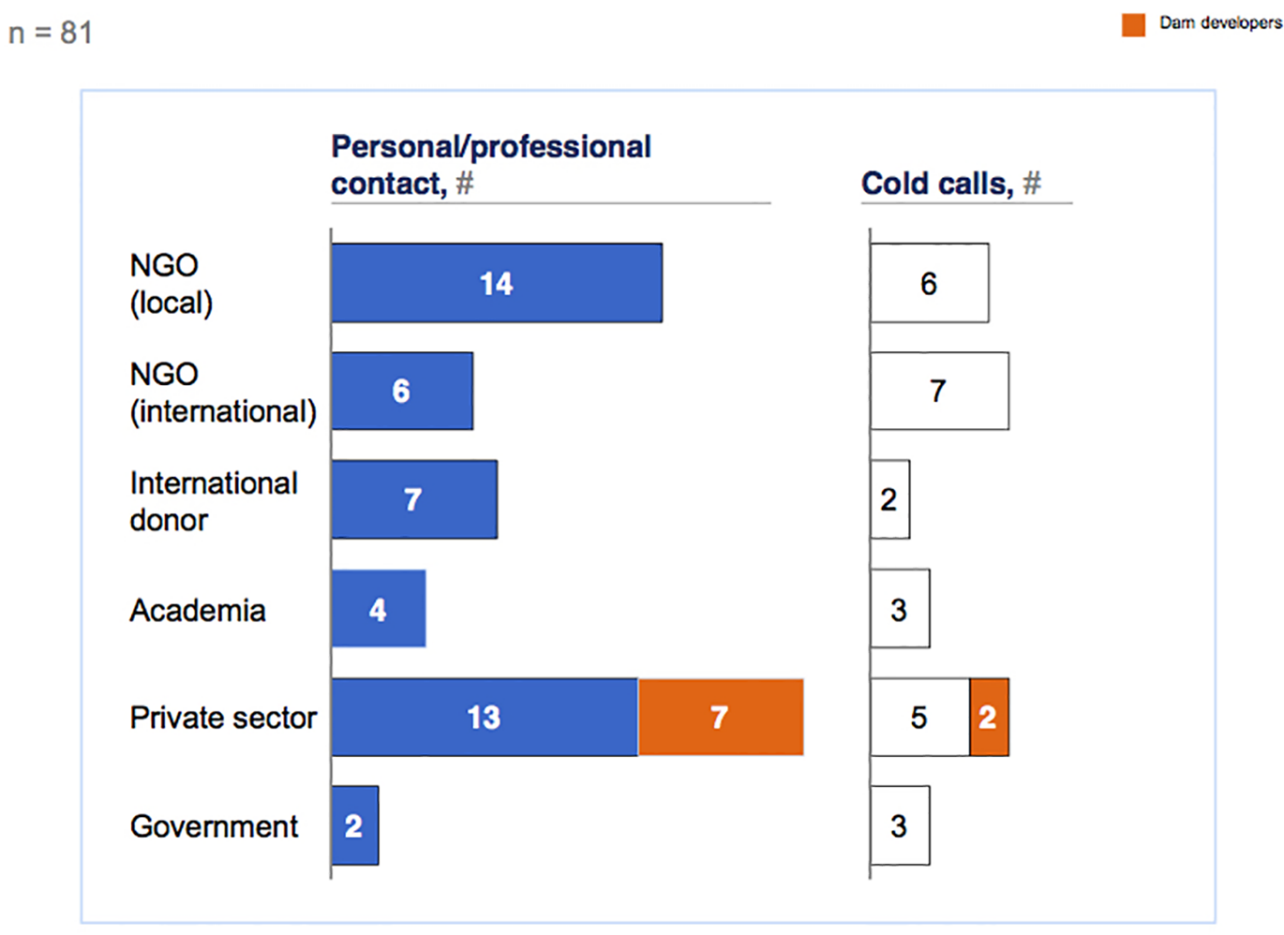
Seeds of completed semi-structured interviews.

Our analysis provides evidence that sample diversity can be reached even if no prior personal or professional contacts to the population of interest have been retained. The seeds of the interviews are depicted in Fig 2, with the left side of the figure depicting the 53 interviews based on a personal or professional contact and the right side depicting the 28 interviews that were based on cold calls. This figure shows two main points of interest: first, both types of seeds include interviews in each interview category; second, the interview sub-category ‘dam developer’, which we hypothesised would be the most difficult to include in the sample, is also covered by both types of seeds. We can therefore conclude that a diverse sample could have been built even if we had relied solely on cold calls.

**Fig 3:**
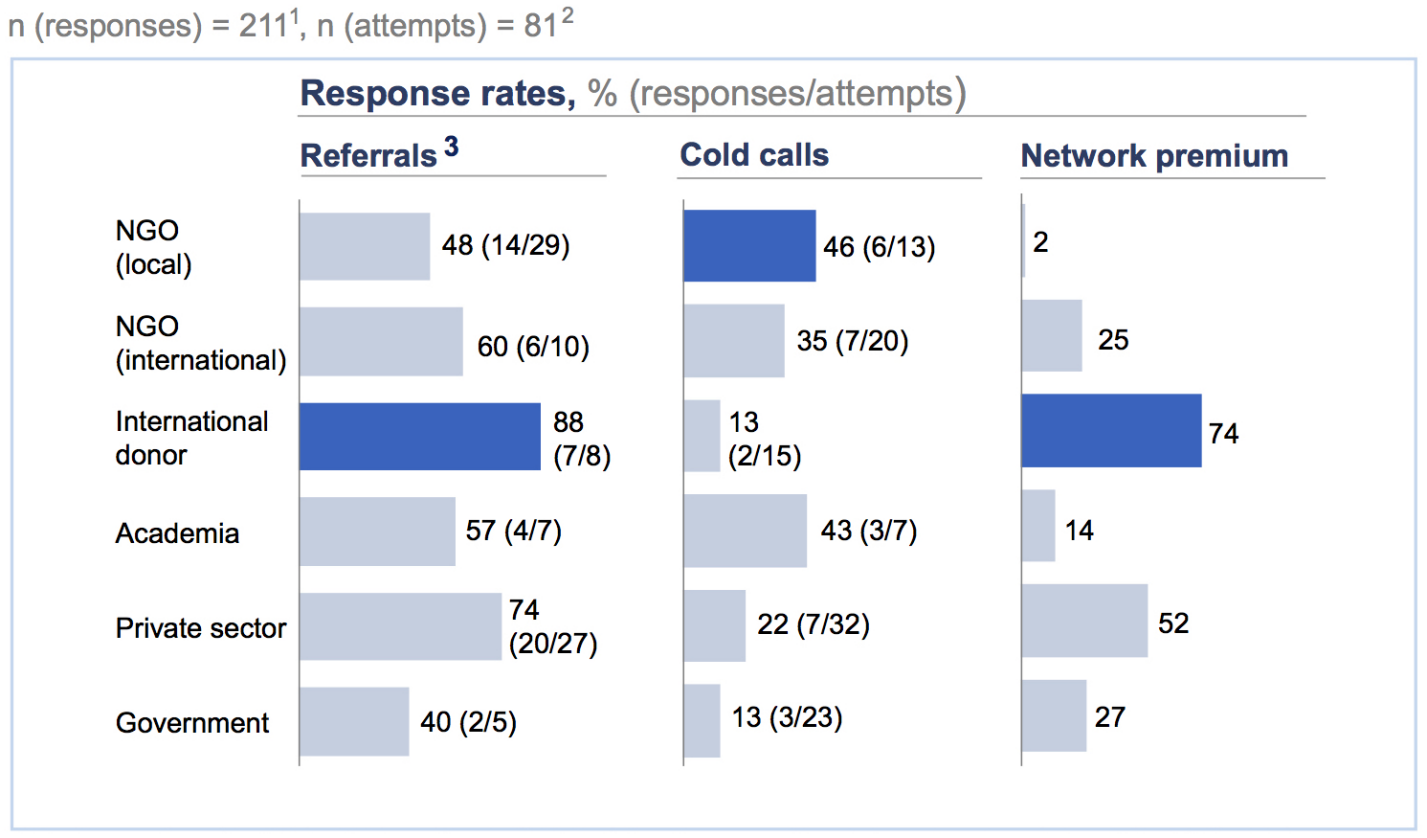
Quantifying the value of referrals. (1) Unable to retrace for 13 identified reach-outs if initiated via referral or cold call; four reach-outs coded as ‘Other’. (2) Unable to retrace for one interview carried out via referral coded as ‘Other’. (3) Including personal contacts and contacts via conferences. (4) Referral response rate–Cold call response rate.

It is acknowledged, however, that building a snowball sample from cold calls is particularly labour-intensive [39]: in our research, only 25% of our cold calls led to an interview, compared to 62% of the referrals. Significant differences in the value of referrals persist from one interviewee group to another (Fig 3). We measure the value of referrals via a concept we call ‘network premium’. To gauge the network premium, we subtracted the cold call response rate (i.e., the number of interviews initiated via cold calls divided by the total number of cold calls) from the referral response rate (i.e. the number of interviews initiated via referrals divided by the total number of referrals). Referrals were the most valuable when contacting international donors and private sector players, with network premiums of 74% and 52%, respectively, indicating that these groups are particularly difficult-to-reach populations.

The overall results from these analyses are encouraging for scholars interested in researching a population to which no personal or professional contacts are retained prior to the research project. While personal or professional contacts maintained to the research population of interest can accelerate the research endeavour, our results also showcase that (at least for our topic of interest) a diverse sample can be built from cold calls if a researcher is willing to invest some time in reach-outs.

### On seed variation

**Fig 4:**
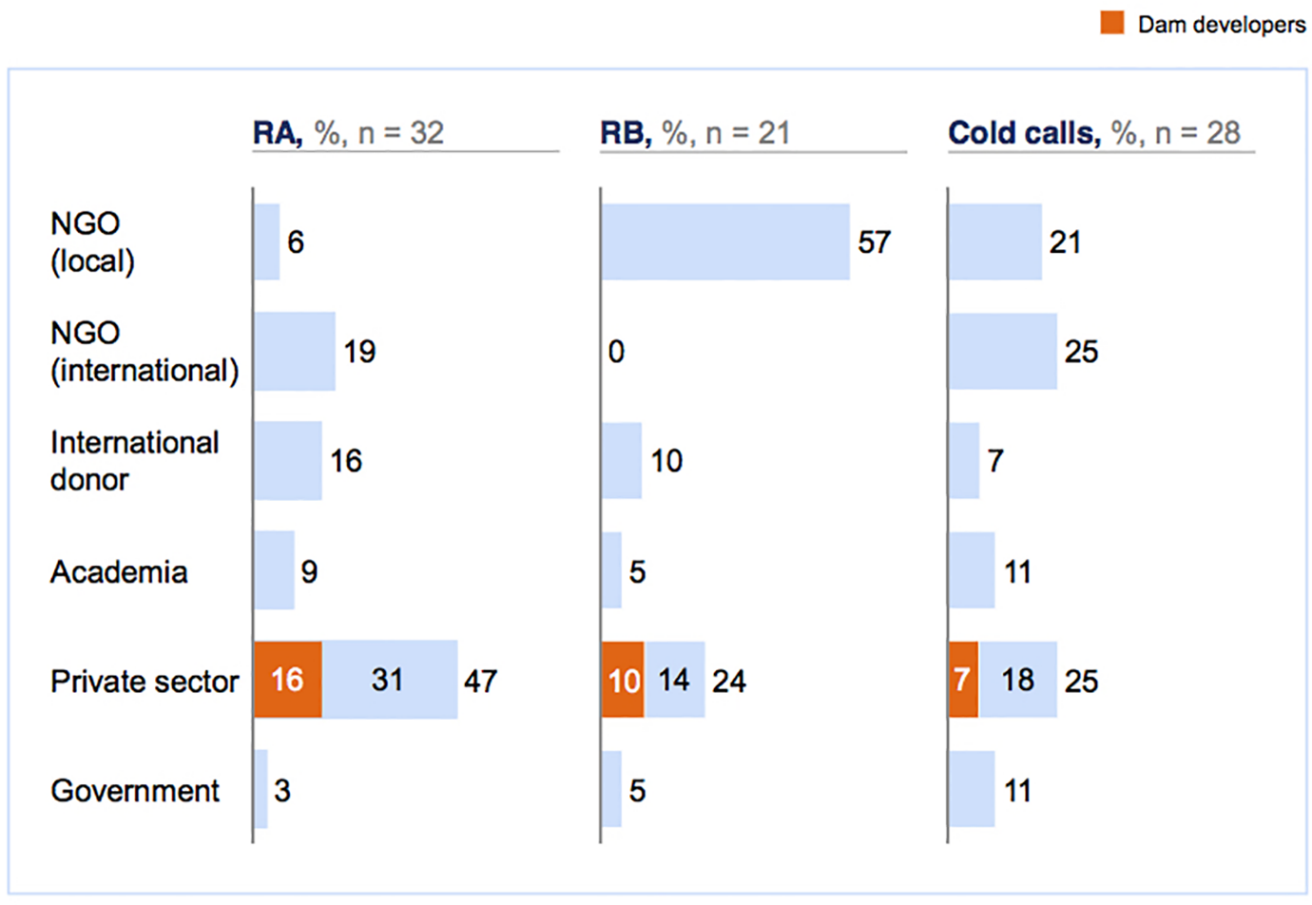
Semi-structured interviews carried out from a seed perspective.

Our research confirms the scholars’ advice that seed diversity is important. Fig 4 (a variation of Fig 2) depicts the completed interviews from a seed perspective, with RA, RB and cold calls as the three main seeds of the sample. The sample built via RA, who has a background in the private sector, is largely biased towards this sector, with 47% of all interviews seeded via RA private sector interviews. RB conducted 57% of interviews, whose background is closest to local NGOs, were with local NGOs. Meanwhile, the sample built via cold calls indicates no significant biases towards any interviewee category. Interviews based on the network of RB included one (TNL17) with a leading activist from a remote area of Myanmar who provided unique insights into the early days of an anti-dam campaign. This insight helped us to develop a narrative of the campaign that was not skewed to the later days of the campaign and the activists prominent in these later days. The sample diversity ensured via RB was thus central to the quality of our research.

It is noteworthy that the three different seeds in Fig 4 include interviews in all interviewee categories, including the sub-category ‘dam developer’ (the sole exception is the interviewee category ‘international NGO, which contains zero interviews for RB). This indicates that, at least for our topic of interest, a fairly diverse sample can be generated even if the researcher is unable to vary her or his seed, although the overall data suggest that seed variation can significantly enhance sample diversity. Fig 3 may therefore be viewed as a case for collaboration among researchers; if researchers with different backgrounds and different personal and professional contacts to the population of interest begin to collaborate, such collaborations are bound to contribute to sample diversity.

### On face-to-face interviews

**Fig 5:**
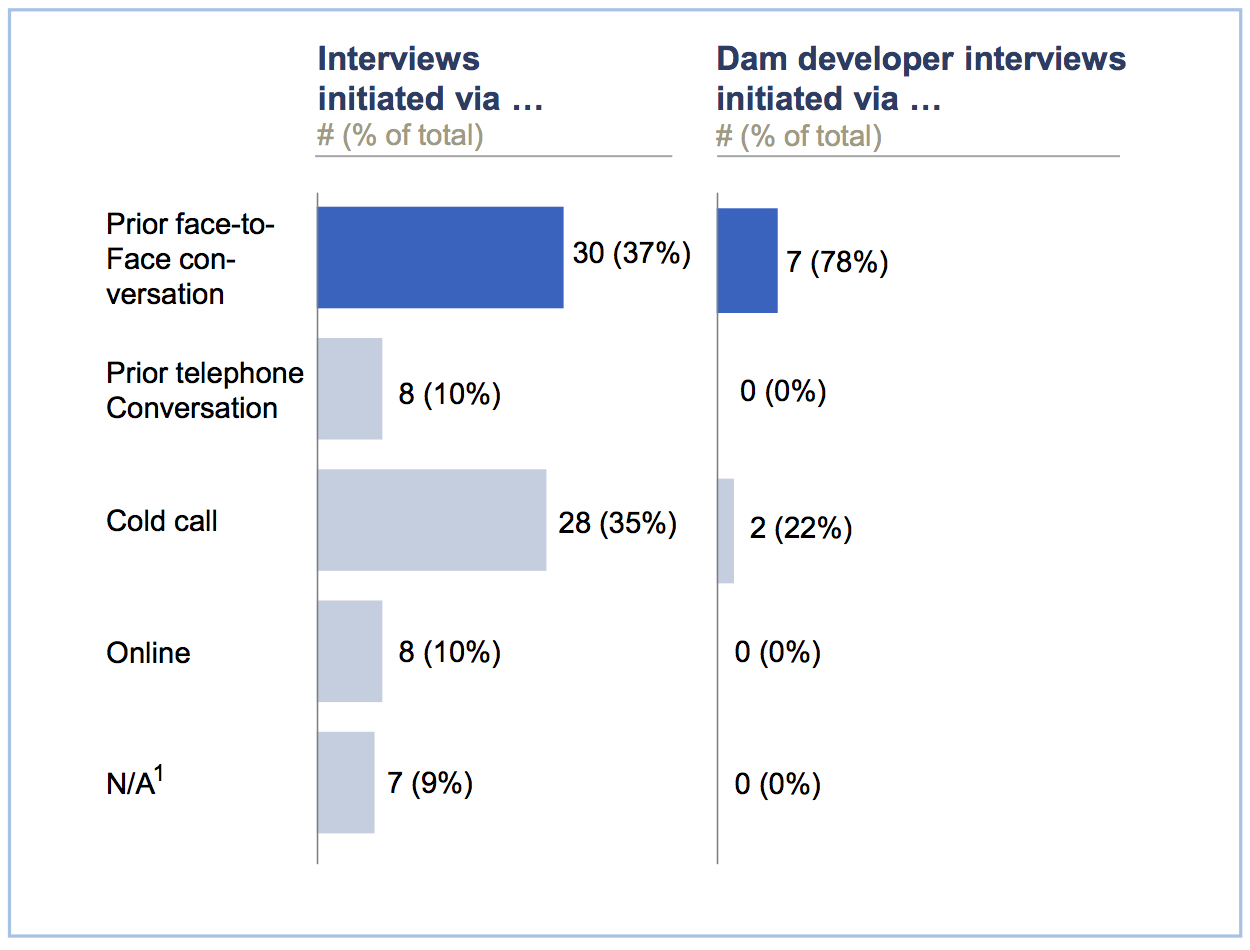
Quantifying the value of face-to-face interviews. (1) Comprises interviews with those already retaining a personal or professional contact prior to the research project.

Our descriptive analysis provides evidence to further support the argument that face-to-face interviews are redundant, with our data indicating that face-to-face interviews can lead to more sought referrals than telephone interviews (perhaps since trust may be more readily established via face-to-face conversations than over the telephone). Fig 5 aims to quantify the value of face-to-face interviews. Overall, 30 (37%) of our interviews were initiated via prior face-to-face conversations, while prior telephone conversations and online contact each led to only eight interviews (10%). An examination shows that of the nine interviews conducted with dam developers, the interviewee sub-category deemed most difficult to access, seven (78%) were initiated via prior face-to-face interviews, while not a single telephone interview led to a referral to a dam developer. These interviews proved to be essential for our research. For instance, one Chinese dam developer challenged a claim from numerous NGOs that his company would not engage with NGOs, which, in turn, allowed us to present a more balanced portrayal of the interplay between Chinese dam developers and NGOs.

While our research did not investigate whether face-to-face interviews lead to lower-quality data than telephone interviews, our data provide tentative evidence that face-to-face interviews are not obsolete; they can still be helpful for those employing or intending to employ snowball sampling, since these interviews can lead to more sought referrals and thus enhanced sample diversity. We acknowledge, however, that this finding may not be true for all populations. For instance, studies on individuals with sexually transmitted diseases have found that these interviewees (particularly men) tend to report more truthfully in an audio-computer-assisted self-interview (ACASI) than in a face-to-face interview, since interviewees tend to be more comfortable reporting on sexually transmitted diseases to a computer than to a live person [81, 82].

### On persistence

**Fig 6:**
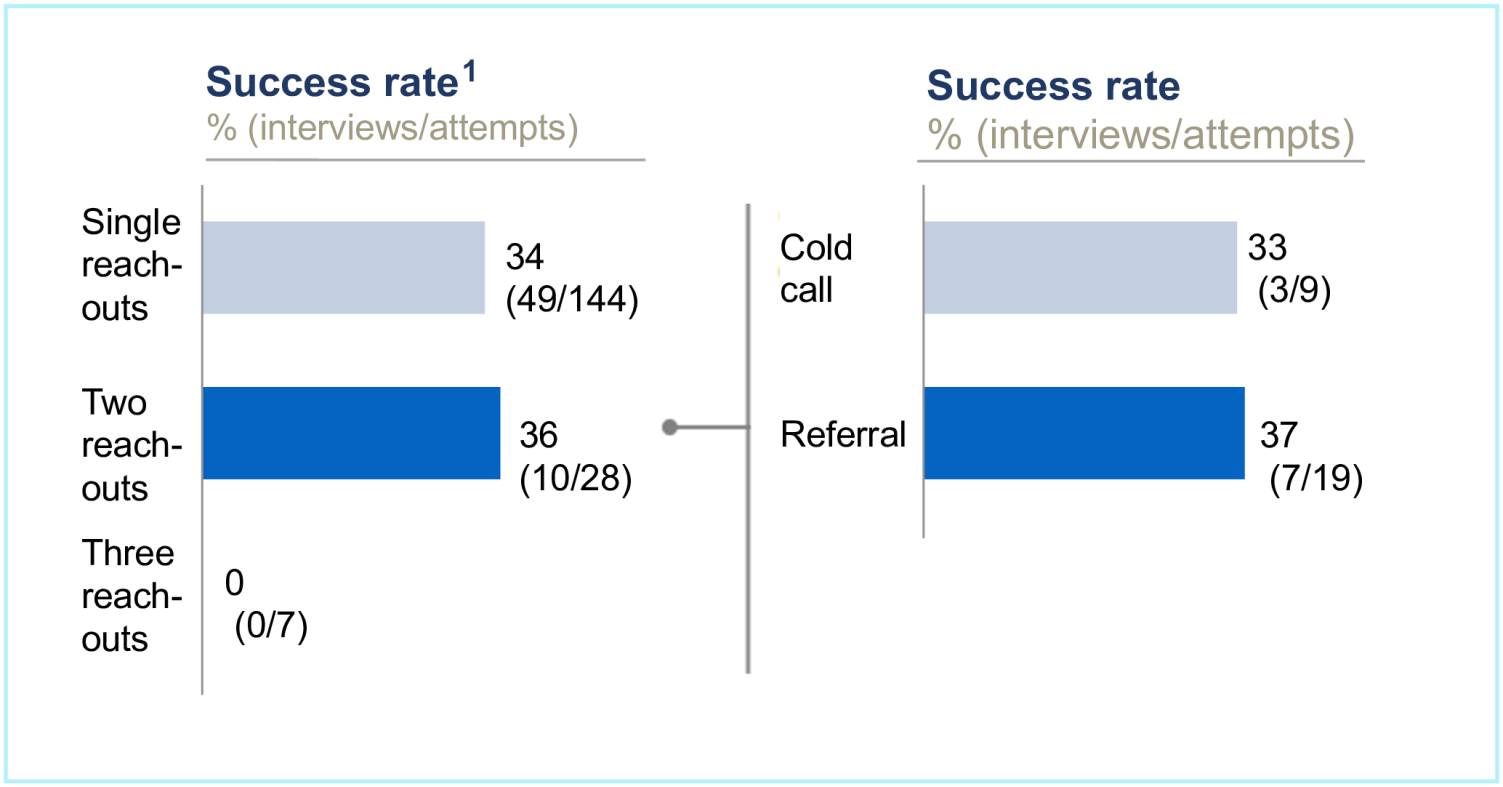
Quantifying the value of persistence. (1) Number of reach-outs unknown for 32 reach-outs. Eight potential interviewees responded, but refused interview.

Our data suggest that persistence can indeed enhance sample diversity, but we can also conclude that excessive persistence does not necessarily yield dividends. Instead of distributing a great many interview reminders during our study, we reached out to the majority of our proposed interview subjects only once. Nevertheless, the scarce data we collected regarding persistence indicates its value. We map this data in Fig 6, with the left side depicting our success rate in relation to the number of reach-outs (either one, two or three) and the right side depicting a deep dive on success rates achieved with two reach-outs (distinguishing between reach-out attempts to unknown potential interviewees and those to whom we were referred by other interviewees). We sent one interview reminder to 28 of our proposed interviewees. This led to 10 additional interviews, a success rate of 36%, equalling 12% of the total interviews analysed for this paper. Reminders appear to be only somewhat more helpful when contacting referrals in comparison to their usefulness with cold calls–a single reminder led to an interview in 39% of our cases for the former group and 38% for the latter. One of the most valuable interviews for our research gained via a reminder was with the CEO of a Burmese dam developer. This interviewee compared Chinese and European dam developers in Myanmar, which helped us to further refine our narrative on social-safeguard policy adherence by Chinese dam developers in Myanmar.

Excessive persistence, however, does not appear to be worthwhile. We sent three reminders to seven of our envisaged interviewees, but as Fig 6 shows, this did not lead to a single additional interview. While our data does not suggest that excessive persistence is helpful to researchers, it may also not be recommended for ethical reasons. A potential interviewee who does not respond to an interview request after two reach-outs may be indicating via this non-response that she or he is not interested in participating in the research. If a single request remains unanswered, the researcher may hypothesise that, for instance, the e-mail was overlooked, a hypothesis particularly likely when conducting interviews with time-pressed leaders of organisations. Indeed, all 10 interviews only carried out upon the second reach-out were interviews with interviewees in management positions.

Our data on persistence provide some evidence that those employing or intending to employ snowball sampling can enhance sample diversity if every reach-out is carefully tracked and followed by a reminder. We typically sent a reminder after one week if no response was obtained upon the first reach-out. This persistence may help to include those least keen to be interviewed for a research endeavour.

### On waves

**Fig 7:**
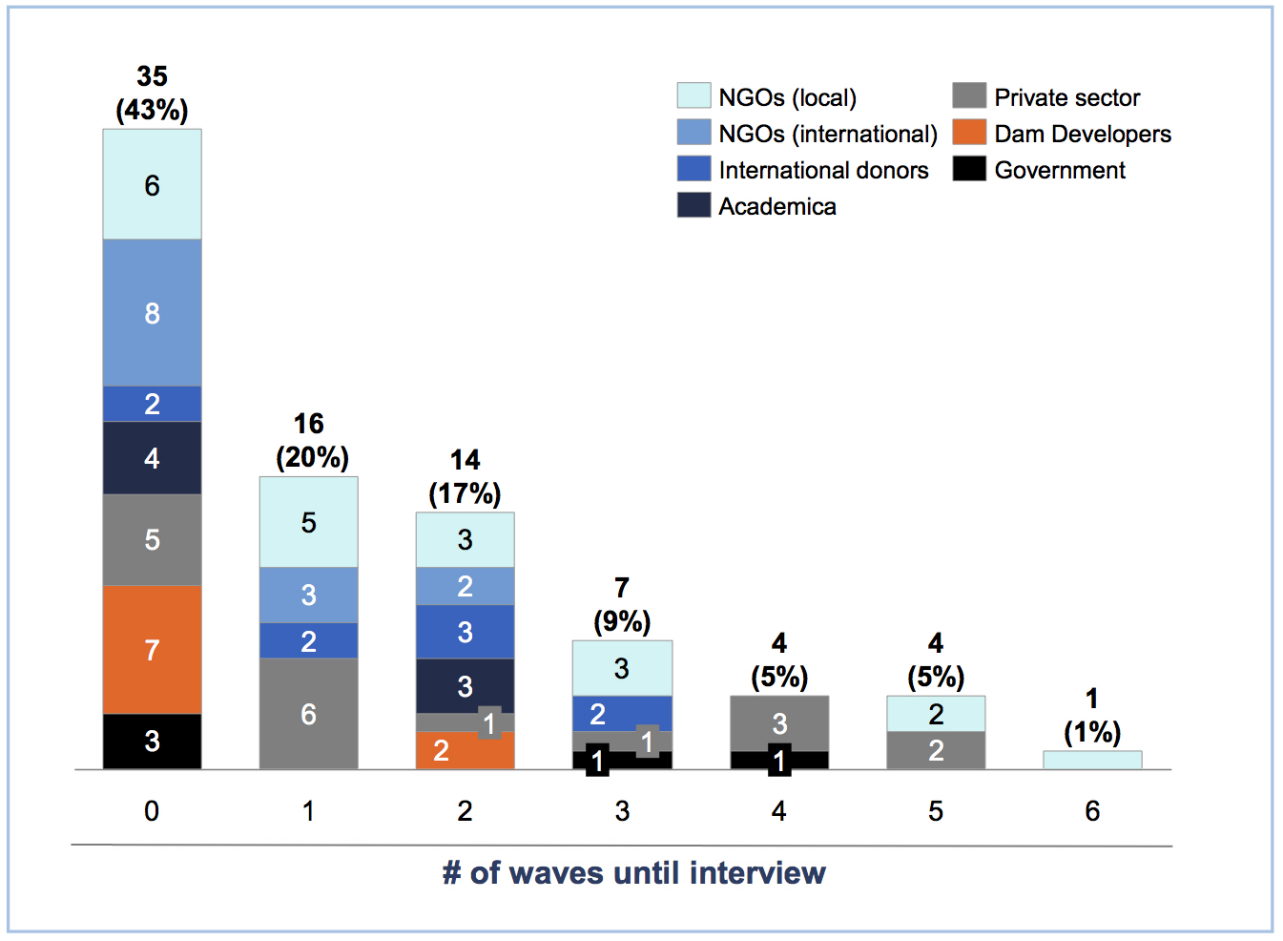
Distribution of sample diversity by sampling.

Our data show some evidence that, for our topic of study, pursuing interviews for even a few waves provided the perspectives of particularly difficult-to-reach populations and thus achieved sample diversity. More than 60% of our interviews were conducted in the zeroth or first wave (Fig 7). These include seven of the nine interviews conducted with dam developers, the sub-category we deemed most challenging to interview. The remaining two interviews with dam developers were conducted in the second wave. However, not a single interview with a dam developer was carried out in the third wave and beyond, although a fifth of our total interviews were carried out in the third or later waves. Pursuing interviews for multiple waves nevertheless yielded novel insights. For instance, interview FNL12, which was conducted in the sixth wave, yielded insights on small dam construction in Myanmar–a topic of (some) interest to our research endeavour, but not covered in detail by previous interviews. Furthermore, we note that our finding regarding the limited value of multiple waves may also be specific to our population, with this finding perhaps indicating a low degree of network segmentation in the population in question [83]. Meanwhile, a high degree of network segmentation may impede the pursuance of multiple waves, since interviewees may lack the suitable contacts for a referral [84].

While additional waves can lead to novel insights, our overall data on waves provide some evidence that the number of waves pursued is not a definitive indicator for sample diversity. Even very few waves can yield access to particularly difficult-to-access populations.

## Conclusion

Our quantitative analysis of pathways to delivering sample diversity in snowball samples yielded the following revisions to the literature’s recommendations:

1. Prior personal contacts are not essential for achieving sample diversity but tend to be helpful, as generating new contacts during research can be labour-intensive.
2. Sample seed diversity is important to achieving sample diversity.
3. Face-to-face interviews build trust and can help to generate further referrals.
4. Persistence (within reason) is helpful in securing interviews.
5. Sample diversity is not necessarily enhanced if a seed is advanced over numerous waves.

We do not claim that these insights are comprehensive, but we believe that these interpretations of our data may serve as a starting point for future scholars using snowball sampling procedures. All of the analyses presented in this section are based only on descriptive statistics. This means, for instance, that we cannot control for confounds such as effort [85]. An experimental research design would yield the most robust insights on sampling procedures to enhance the sampling diversity of a snowball sample (with, for instance, one research project staffed with scholars with relevant personal or professional contacts and another staffed with scholars without relevant contacts).

Overall, this work aims to advance the literature on snowball sampling as a qualitative sampling approach. While snowball sampling procedures may qualify ‘as the least “sexy” facet of qualitative research’ ([1], p. 328), these procedures are ‘not self-evident or obvious’ ([20], p. 141), since the snowball sample does not ‘somehow magically’ ([20], p. 143) start, proceed and terminate when a scholar attempts to develop a diverse sample. Rather, continuous, deliberate effort by the researcher(s) is required. Our paper has attempted to provide some insights on this effort.

Unfortunately, we developed the idea to write this paper only during the course of our research project, and thus some of our data may be skewed. For instance, we may not have been able to trace all original reach-out attempts and our data on persistence may therefore be biased. Some of those scholars grounded in quantitative thinking may also claim that the insights outlined in Section 4 lack external validity since our sample size is relatively small from a quantitative methodological perspective. In addition, our population was very specific and thus may not be comparable to other difficult-to-reach populations, and we also did not adopt an experimental research design as described above. Hence, we encourage scholars to replicate our findings via their respective research projects that employ snowball sampling. With many scholars claiming to feel more pressed than ever to deliver research results with maximum efficiency, we hope that these initial descriptive analyses of snowball sampling procedures provide some valuable insights to those employing or intending to employ this method and aiming to improve their management of it.

## Supporting information

S1 File (XLSX)
